# The Efficacy of Short-Duration Polyethylene Glycol plus Electrolytes for Improving Bowel Preparation of Colonoscopy in Patients with Chronic Constipation

**DOI:** 10.1155/2020/8886073

**Published:** 2020-11-24

**Authors:** Naohisa Yoshida, Yoshikazu Inagaki, Kohei Fukumoto, Hiroyuki Yoriki, Yutaka Inada, Takaaki Murakami, Yuri Tomita, Hikaru Hashimoto, Satoshi Sugino, Ryohei Hirose, Osamu Dohi, Ken Inoue, Yoshito Itoh

**Affiliations:** ^1^Department of Molecular Gastroenterology and Hepatology, Kyoto Prefectural University of Medicine, Graduate School of Medical Science, Kyoto, Japan; ^2^Department of Gastroenterology, Nishijin Hospital, Kyoto, Japan; ^3^Department of Gastroenterology, Nara City Hospital, Nara, Japan; ^4^Department of Gastroenterology, Otsu City Hospital, Shiga, Japan; ^5^Department of Gastroenterology, Kyoto First Red Cross Hospital, Kyoto, Japan; ^6^Department of Gastroenterology, Aiseikai Yamashina Hospital, Kyoto, Japan

## Abstract

**Materials and Methods:**

This multicenter retrospective study was conducted from September 2019 to September 2020 at 5 related institutions among patients ≥ 20 years old diagnosed with chronic constipation whose previous colonoscopic BP had had a fair or poor Aronchick score. Two or four sachets of PEG+E (13.7 or 27.4 g/day) were prescribed for 1 week before colonoscopy. We analyzed the rate of improvement in BP, effect-related factors, spontaneous bowel movements (SBMs), stool consistency, improvement of constipation symptoms, and adverse events.

**Results:**

We evaluated 106 cases (56 males) with an average age of 69.5 ± 9.4 years old (≤74 years old: 68 cases, ≥75 years old: 38 cases). The improvement rate of BP was 72.6%, and the insertion time and pain score also improved. A performance status of 1 or 2 was associated with poor BP. SBMs (times/week) increased from 4.0 ± 1.9 to 6.1 ± 2.6 (*p* < 0.001). The overall improvement rates of SBMs, stool consistency, symptoms of constipation, and rate of adverse events were 58.5%, 90.6%, 59.4%, and 6.6%, respectively, showing no significant differences with regard to age or gender.

**Conclusions:**

Short-duration PEG+E was effective for improving poor BP and chronic constipation.

## 1. Introduction

Colonoscopy is widely performed for screening, surveillance after polyp and cancer resection, various abdominal complaints, and removal of polyps and cancers. Resection of polyps is reported to lead to a reduction in colorectal cancer death [1]. However, up to 30% of colonoscopies have poor bowel preparation (BP), which leads to decreased lesion detection due to poor visualization and an increased need for repeat colonoscopies [2, 3]. An older age, male sex, inpatient status, diabetes mellitus, constipation, and tricyclic antidepressant use are known to be associated with inadequate BP [4]. In particular, constipation increases the risk of inadequate BP about twofold [5, 6]. For cases with these risk factors, we regularly strengthen the method of BP, such as increasing the amount of polyethylene glycol (PEG), adding a laxative before the procedure, and performing strict diet limitation [7]. Regarding the addition of a laxative, bisacodyl, sennoside, picosulfate sodium, magnesium oxide, and short-duration PEG are prescribed in clinical practice. However, no evidence concerning the efficacy of these additional laxatives for this purpose has yet been obtained.

In Japan, magnesium oxide and anthraquinolone stimulant laxatives (e.g., sennoside) have been widely used for chronic constipation. The guideline for the medical treatment of constipation set by the American College of Gastroenterology proposes lifestyle habit guidance and the administration of osmotic laxatives [8–10]. In 2018, sachets of an osmotic laxative of PEG4000 plus electrolytes (PEG+E: Movicol: EA Pharma, Tokyo, Japan) were launched for chronic constipation in Japan, and the recent Japanese guideline now recommends this drug instead of anthraquinolone stimulant laxatives [11]. It is minimally absorbed and increases the water content of stool in a dose-dependent manner. Previous reports about the efficacy of this drug are limited for nonelderly people and female [12–14].

In the present study, we analyzed the efficacy of short-duration PEG+E for chronic constipation and improving poor BP for colonoscopy in patients with chronic constipation, including male gender and elderly people.

## 2. Patients and Methods

This was a multicenter, single-arm, retrospective cohort study. We reviewed 118 patients from 5 related institutions diagnosed from September 2019 to September 2020 with chronic constipation whose previous colonoscopic BP had been fair or poor on the Aronchick bowel preparation scale [15]. The related institutions were Kyoto Prefectural University of Medicine, Nishijin Hospital, Otsu City Hospital, Nara City Hospital, and Aiseikai Yamashina Hospital.

The inclusion criteria were patients ≥ 20 years old suffering from ≥2 of the following 6 criteria of chronic constipation under the Rome IV standard: straining, hard stool, residual stool feeling, occlusion feeling, manual bowel movement (BM) performed ≥25% of overall BMs, and BM frequency < 3 times a week [16]. A chronic status was defined as symptoms being present for at least six months, with the symptoms described above being present for at least three months. The diagnosis of chronic constipation was made by each doctor in each institution, and the study representative endoscopist (N.Y.) reconfirmed whether or not the definition had been met in each case. We excluded patients with fatal cardiopulmonary, hepatic, or renal disease. We excluded cases with ≥7 BMs/week who met the definition of chronic constipation because these cases might have had irritable bowel syndrome. Short-duration PEG+E (6.8 g/sachet) at 13.7 g/day was prescribed initially 1 week before colonoscopy. The PEG+E was dissolved in 125 mL of water. After 2 days' intake, the amount of PEG could be increased to 27.4 g according to the stool frequency and consistency.

The evaluation items for this study were the patients' characteristics, improvement in colonoscopic BP after PEG+E, and efficacy of PEG+E for chronic constipation. The improvement in the BP was defined as an increase of at least 1 score in the Aronchick score. We divided all cases into improved and nonimproved BP groups and analyzed the colonoscopic status and effect-related factors among patient characteristics as well as the underlying disease and concomitant medications. The colonoscopic status included the rate of cecal intubation, insertion time, and pain score. The pain score was scored as 0 (no pain), 1 (mild pain), 2 (moderate pain), or 3 (severe pain) by each operator. Regarding the efficacy of PEG+E for chronic constipation, we analyzed the number of spontaneous BMs (SBMs) 1 week before and after the administration of PEG+E according to the number of previous BMs (<3/week or 3-6/week), gender (male or female), and age (≤74 or ≥75 years old). The number of SBMs referred to BMs that occurred without a laxative/enema or manual evacuation. The improvement rate for SBM was also calculated according to the number of previous BMs, gender, and age and defined as an increase in ≥1 BM per week from the baseline with ≥3 BMs/week, in reference to a previous report [17].

The changes in stool consistency according to the Bristol stool form scale (BSFS) were also analyzed according to previous number of BMs, gender, and age [18]. The BSFS is a global standard for the evaluation of the stool shape (range of 1 to 7). Types 1 and 2 are hard stools, types 3-5 are normal stools, and types 6 and 7 are loose stools. With respect to the stool consistency, an “increase to types 3-5” was considered as a sign of improvement. The improvement rate of constipation symptoms, such as straining, residual stool feeling, and occlusion feeling, was also analyzed. The time until the first SBM after the administration of PEG within 48 h and the rate of SBM within 24 h were also analyzed. Adverse events were examined according to the previous number of BMs, gender, and age.

With respect to colonoscopic BP, we used highly concentrated PEG (MOVIPREP; EA Pharma, Tokyo, Japan) according to our previous report [19]. In brief, patients received a low residual diet on the day before colonoscopy and consumed 10 mL of picosulfate sodium at 9-10 PM that same day. Patients then took 1.0 L of highly concentrated PEG and 0.5 L of water 3 h prior to the examination on the day of colonoscopy. All colonoscopies were performed by 5 veteran endoscopists who had experience performing more than 5,000 colonoscopies.

We obtained informed consent from all patients before the colonoscopy. This study was retrospective in setting, and an opt-out about the study to the patients was performed in the representative facility (Nishijin Hospital). This research was approved by the Ethics Committee of Nishijin Hospital (Number 20-05, approved data: May 14, 2020) and Kyoto Prefectural University of Medicine (ERB-C-1600, approved data: Dec. 23, 2019) and was in accordance with the World Medical Association Declaration of Helsinki.

### 2.1. Statistical Analyses

The Mann-Whitney *U* test, chi-squared test, and Yates continuity correction were used in this study. To compare continuous variables, the Mann-Whitney *U* test was used. Categorized variables were analyzed by the chi-square test and Yates continuity correction. All statistical analyses were performed using the SPSS software program (IBM Japan, Ltd., Tokyo, Japan). *p* < 0.05 was considered significant for all statistical analyses.

## 3. Results

**Figure 1:**
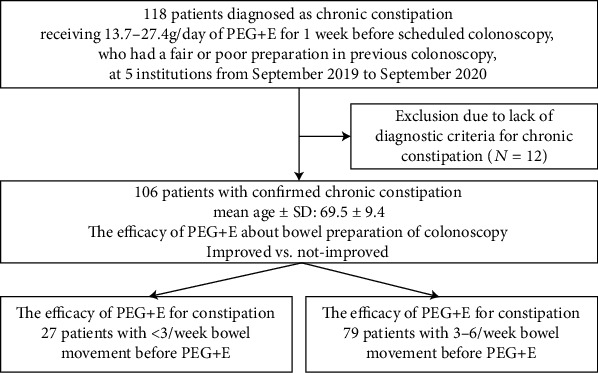
A flow diagram of the present study. PEG+E: polyethylene glycol plus electrolytes; SD: standard deviation.

After excluding 12 cases that did not meet the diagnostic criteria for chronic constipation, we finally analyzed 106 cases with chronic constipation to determine the improvement in BP and chronic constipation (Figure 1). The gender was male in 56 (52.8%) and female in 50 (47.2%) with an average age of 69.5 ± 9.4 years old. Regarding the age distribution, 38 cases (35.8%) were ≥75 years old (Table 1). Thirty-five cases (33.0%) had used other laxatives. The final dose of PEG+E was 13.7 g/day for 94 cases (88.7%) and 27.4 g/day for 12 cases (11.3%). With respect to the definition of chronic constipation, 27 cases (25.5%) had <3 BMs/week, 60 cases (56.6%) had hard stool (Bristol bowel consistency scale 1 or 2), and 99 cases (93.4%) had other symptoms of constipation according to the Rome IV criteria, such as straining, residual stool feeling, and occlusion feeling.

**Figure 2:**
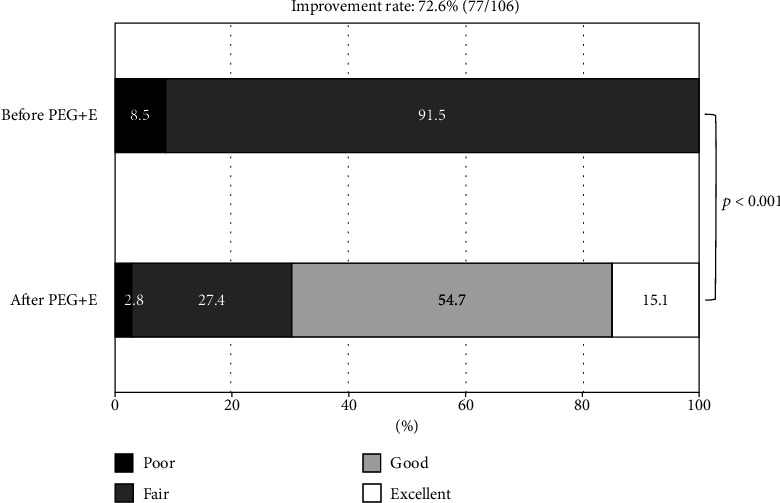
Improvement rate of bowel preparation after prescription of short-duration PEG+E. PEG+E: polyethylene glycol plus electrolytes.

With respect to the improvement rate in the frequency of SBMs, 77 cases showed efficacy (72.6%), with their score improving by 1 to 2 scores (improvement of 2 scores in 18 cases, improvement of 1 score in 59 cases; Figure 2). The rate of excellent+good was 69.8%.

A comparison between the improved BP and the non-improved BP group was made (Table 2). There were no significant differences with respect to the gender, age, body mass index, concomitant use of laxatives, dose of PEG, presence of various underlying diseases, and use of concomitant drugs. The rate of a poor performance status (scale 1+2) was higher in the non-improved BP group than in the improved BP group (27.6% vs. 7.8%, *p* = 0.01). Regarding colonoscopy, the insertion time (min, mean ± standard deviation (SD)) was shorter in the improved BP group than in the non-improved BP group (8.3 ± 6.4 vs. 9.2 ± 7.5, *p* = 0.03). The pain score (mean ± SD) was also better in the improved BP group than in the non-improved BP group (0.4 ± 0.8 vs. 0.6 ± 0.9, *p* = 0.02).

**Figure 3:**
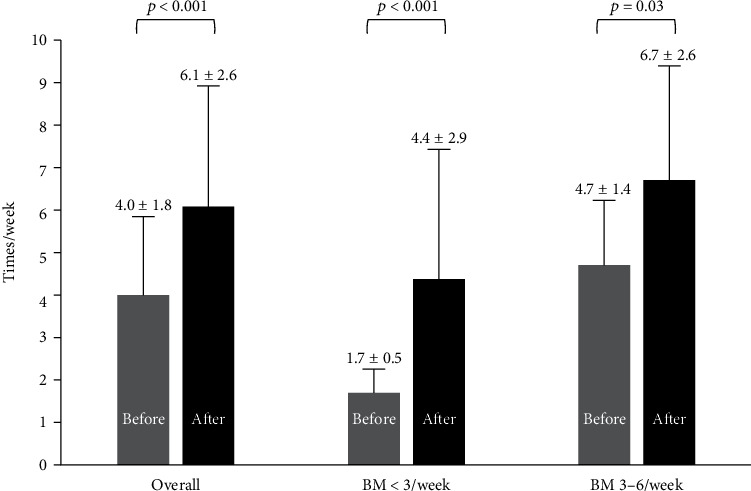
Changes of spontaneous bowel movements before and after short-duration PEG+E both for those with <3 BMs/week and 3-6 BMs/week. PEG+E: polyethylene glycol plus electrolytes; BM: spontaneous bowel movement.

The frequency of SBMs (times/week, average ± SD) in the week after PEG+E administration significantly improved compared with that before its administration (6.1 ± 1.8 vs. 4.0 ± 1.8, *p* < 0.001) (Figure 3). The frequency of SBM (times/week) increased from 1.7 ± 0.5 to 4.4 ± 2.9 in 27 cases with <3 SBMs/week (*p* < 0.001) and from 4.7 ± 1.4 to 6.7 ± 2.6 times/week in 79 cases with 3-6 SBMs/week (*p* = 0.03).

The improvement rates of SBMs/week, stool consistency, and constipation symptoms were 58.5% (62 cases), 90.6% (96 cases), and 59.4% (63 cases), respectively (Table 3). The improvement rates of SBMs/week in cases with <3 BMs/week and 3-6 BMs/week were 77.8% and 51.9%, respectively (*p* = 0.01). There were no significant differences in these rates, regardless of gender and age.

The mean time to first SBM within 48 h after taking PEG+E was 25.7 ± 10.1 h (Table 4). This time was significantly longer for those with <3 BMs/week than for those with 3-6 BMs/week (29.7 ± 13.0 vs. 24.6 ± 9.0, *p* = 0.01). Regarding gender and age, there were no significant differences in this time. The SBM rate within 24 h after taking PEG+E was 82.1% (87 cases); it was 69.6% for those with <3 BMs/week and 89.9% for those with 3-6 BMs/week (*p* = 0.03). Regarding gender and age, there were no significant differences in the rate.

Adverse events were observed in 7 cases (6.6%), as follows: 2 cases (1.9%) of abdominal pain, 2 cases (1.9%) of increase residual stool feeling, 1 case (0.9%) of diarrhea, 1 case (0.9%) of abdominal distension, and 1 case (0.9%) of abdominal discomfort (Table 5). Regarding the number of BMs before prescription of PEG-E, gender, and age, the rates of adverse events were 7.4% (2 cases: 1 abdominal pain and 1 diarrhea) in those with <3 BMs/week and 6.3% (5 cases: 1 abdominal pain, 2 increase of residual stool feeling, 1 abdominal distension, and 1 abdominal discomfort) in those with 3-6 BMs/week (*p* = 0.94), 7.1% (4 cases: 2 increase residual stool feeling, 1 abdominal distention, and 1 abdominal discomfort) in males and 6.0% (3 cases: 2 abdominal pain and 1 diarrhea) in females (*p* = 0.87), and 8.8% (6 cases, 1 abdominal pain, 2 increase residual stool feeling, 1 diarrhea, 1 abdominal distension, and 1 abdominal discomfort) in those ≤74 years old and 2.6% (1 case: 1 abdominal pain) in those ≥75 years old (*p* = 0.21).

## 4. Discussion

In this study, we showed a 72.6% improvement rate for poor colonoscopic BP using short-duration prescription of PEG+E, an efficacy that was achieved regardless of gender, age, underlying diseases, and concomitant prescription, including laxatives—but not a poor performance status. This is the first report describing the efficacy of additional daily PEG+E prior to colonoscopy for improving poor colonoscopic BP. Additional treatments typically given on the day of colonoscopy for poor BP can be slightly invasive, such as an enema or increase in cleansing solution. Instead of these treatments, we suggest daily short-duration PEG+E as a less-invasive approach. In addition, this improvement in BP resulted in a shorter insertion time and lower pain score, and improved BP is also reported to yield an increase of adenoma detection rate and decrease in missed polyps [3].

Previous studies have described the efficacy of PEG for chronic constipation [17–21]. In a randomized control trial (RCT) comparing PEG3350+E to placebo from the UK, the number of cases in the PEG3350+E group, their mean age, and the rate of female gender were 68 cases, 43.6 ± 14.9 years old, and 85.1%, respectively. The number of SBMs/week in the PEG group taking PEG for 4 weeks was 4.40 ± 2.58 compared to 3.11 ± 1.93 in the placebo group (*p* < 0.00001) [17]. A recent Japanese RCT using the same dose of PEG3350+E as our study involved 80 patients taking PEG+E, with a mean age of 44.3 ± 11.6 years old and 88.8% females [18]. The baseline number of SBMs/week was 1.6 ± 0.9, which increased during the first and second week of PEG3350+E prescription to 3.36 (95% confidence interval (CI): 2.81-3.92) and 4.27 (95% CI: 3.36-4.92), respectively, with responder rates of 80.0% and 86.3%, respectively. These two studies could not include enough elderly people and male gender. In our study, the mean age and the rate of females were 69.5 ± 9.4 and 47.2% so that we could show the efficacy of PEG+E for elderly people and male gender. In addition, the overall baseline number of SBMs/week (4.0 ± 1.8) was higher than in the present study; although, the overall improvement rate of SBM (58.5%) was lower. However, the baseline number of SBMs/week, SBMs/week after PEG+E, and improvement rate of SBM in patients with <3 BMs/week in the present study were 1.7 ± 0.5, 4.4 ± 2.9, and 77.8%, respectively, and these results were comparable to those of the two previous studies. We suggested our study included more moderate cases than the previous studies, suggesting that PEG+E might be less effective for increasing SBM in patients with moderate constipation than severe constipation. In addition, our study included more males than the previous studies, and the efficacy of PEG+E for males seemed slightly lower than that for females.

Regarding stool consistency, in the previous study, the rates of improvement in stool consistency (BSFS 3, 4, and 5) at the first and second weeks were 87.3% and 80.0%, respectively, which was comparable to that in our study (90.6%) [18]. The authors analyzed the rates of complete SBMs, defined as SBMs with a feeling of complete evacuation, and the rates in the first and second weeks were 23.8% and 37.5%, respectively. We calculated the improvement rate in overall constipation symptoms instead of complete SBMs and found this rate to be 59.4%. This difference was due to the high rate of cases with 3-6 BMs/week, as the improvement rate about symptoms in those cases was higher than in cases with <3 BMs/week.

In an RCT from France, comparing PEG4000 to lactulose, the case number, rate of females, and mean age in the PEG group were 32, 84.3%, and 57 ± 19 years old, and the average amount of PEG4000 was 19 ± 5 g/day [19]. The rates of improvement in stool frequency from <3/week, difficulty in defecating, and a straining feeling were 62%, 69%, and 37%. These rates were comparable to those in our study, although the ethnicity and rate of females were different to our study.

Another paper also suggested that PEG was not markedly affected by any ethnic factors because it is mostly not absorbed and only increases the water content of stool in a dose-dependent manner [20]. Regarding age, we compared the efficacy in patients ≤ 74 years old to that in patients ≥ 75 years old, noting no significant difference. In children, a systematic review of three studies showed better results with regard to the number of SBMs for PEG than for lactulose [21]. We therefore suggest that PEG+E is effective for increasing the SBM, regardless of age.

Regarding adverse events due to the drug, the rate was 7.5% (6/80) in a previous Japanese study, all of which were mild gastrointestinal disorders, including abdominal pain and diarrhea [18]. The rate in a study from France was 15.7% (5/32), all of which were mild abdominal pain and distention [20]. In addition, the rate in a report from the UK was 9.0%, including abdominal pain in 4.5% and diarrhea in 4.5% [17]. The rate in the present study was 6.6%, with no significant differences noted in the baseline number of BMs, gender, or age. We demonstrated the high safety of a small amount of PEG+E for chronic constipation.

This study was limited by its retrospective nature and small number of cases. Thus, there was a selection bias about enrolled patients because it was not consecutive and decided by each doctor's decision.

## 5. Conclusion

In more than 100 clinical cases, including elderly and male patients, short-duration and small-amount PEG+E was effective for improving poor colonoscopic BP, SBMs, stool consistency, and symptoms of constipation, regardless of age and gender.

## Figures and Tables

**Table 1:** Patients' characteristics.

	106
Gender, *n* (%); male : female	56 : 50 (52.8 : 47.2)
Age, mean ± SD	69.5 ± 9.4
Age distribution, *n* (%) ≤74 : ≥75 years old	68 : 38 (64.2 : 35.8)
Body mass index, mean ± SD	23.3 ± 3.9
Performance status (0 : 1 : 2), *n* (%)	92 : 10 : 4 (88.8 : 9.3 : 1.9)
Prescription of laxative, *n* (%)	35 (33.0)
Prescription of irritant laxative, *n* (%)	23 (21.7)
Dose of PEG-E/day, *n* (%) 13.7 g : 27.4 g	94 : 12 (88.7 : 11.3)
Definition of chronic constipation	
<3 BMs	27 (25.5)
Bristol bowel consistency scale 1 and 2	60 (56.6)
Symptoms besides BM and bowel consistency	99 (93.4)
Underlying disease	
Hyperlipidemia, *n* (%)	32 (30.2)
Colorectal surgery, *n* (%)	16 (15.1)
Diabetes, *n* (%)	13 (11.2)
Hypothyroidism, *n* (%)	5 (5.6)
Hepatic or biliary disorder, *n* (%)	4 (3.7)
Parkinson's disease, *n* (%)	1 (0.9)
Concomitant medication	
Antacids, *n* (%)	28 (23.4)
Calcium antagonists, *n* (%)	28 (23.4)
Antidepressants, *n* (%)	14 (7.5)
Opioids, *n* (%)	0 (0.0)

SD: standard deviation; PEG+E: polyethylene glycol plus electrolytes; BM: bowel movement.

**Table 2:** The comparison between cases with and without improvement of bowel preparation.

	Improved BP	Non-improved BP	*p* value
Case number	77 (72.6)	29 (27.4)	
Gender, *n* (%), male : female	40 : 37 (51.9 : 48.1)	16 : 13 (55.2 : 44.8)	0.77
Age, mean ± SD	68.7 ± 9.8	71.6 ± 8.6	0.08
Age, *n* (%), ≤74 : ≥75 years old	50 : 27 (64.9 : 35.1)	18 : 11 (62.1 : 37.9)	0.94
Body mass index, mean ± SD	23.2 ± 3.8	23.6 ± 4.2	0.36
Performance status (0 : 1 + 2), *n* (%)	71 : 6 (92.2 : 7.8)	21 : 8 (72.4 : 27.6)	0.01
Laxative combination, *n* (%) Irritant laxative, *n* (%)	25 (32.5) 14 (18.2)	10 (44.4) 9 (29.6)	0.84 0.15
Dose of PEG/day (13.7 g : 27.4 g), *n* (%)	68 : 9 (88.3 : 11.7)	26 : 3 (89.7 : 10.3)	0.88
Colonoscopy			
Cecal intubation, *n* (%)	77 (100)	29 (100)	1.0
Insertion time (min), mean ± SD	8.3 ± 6.4	9.2 ± 7.5	0.03
Pain score^∗^, mean ± SD	0.4 ± 0.8	0.6 ± 0.9	0.02
Underlying disease			
Hyperlipidemia, *n* (%)	23 (29.9)	9 (31.0)	0.90
Colorectal surgery, *n* (%)	14 (18.2)	2 (6.9)	0.25
Diabetes	10 (13.0)	3 (10.3)	0.97
Hypothyroidism	4 (5.2)	1 (3.4)	0.89
Hepatic or biliary disorder	4 (5.2)	0 (0.0)	0.49
Parkinson's disease	0 (0.0)	1 (3.4)	0.60
Concomitant medication			
Antacids, *n* (%)	20 (26.0)	8 (27.6)	0.86
Calcium antagonists, *n* (%)	21 (27.3)	7 (24.1)	0.74
Antidepressants, *n* (%)	8 (10.4)	6 (20.7)	0.28

BP: bowel preparation; SD: standard deviation; PEG+E: polyethylene glycol plus electrolytes; pain score (0: no pain, 1: minimum pain, 2: moderate pain, and 3: severe pain).

**Table 3:** Improvement of SBMs, stool consistency, and constipation symptoms in number of BMs/week, gender, and age.

	Case number	Improvement rate of SBMs/week	*p* value	Improvement rate of stool consistency	*p* value	Improvement rate of constipation symptoms	*p* value
Overall, *n* (%)	106	62 (58.5)		96 (90.6)		63 (59.4)	
<3 BMs/week, *n* (%)	27	21 (77.8)	0.01	24 (88.9)	0.72	12 (44.4)	0.06
3-6 BMs/week, *n* (%)	79	41 (51.9)		72 (91.1)		51 (64.6)	
Male, *n* (%)	56	30 (53.6)	0.27	50 (89.3)	0.63	33 (58.9)	0.91
Female, *n* (%)	50	32 (64.0)		46 (92.0)		30 (60.0)	
≤74 years old, *n* (%)	68	37 (54.4)	0.25	60 (88.2)	0.27	43 (63.2)	0.28
≥75 years old, *n* (%)	38	25 (65.8)		36 (94.7)		20 (52.6)	

SBM: spontaneous bowel movement; BM: bowel movement.

**Table 4:** Time to first SBMs and rate of SBMs within 24 h after prescription of short-duration PEG+E.

	Time to first spontaneous BMs within 48 h, mean ± SD (*n*)	*p* value	Rate of spontaneous BMs within 24 h, % (*n*)	*p* value
Overall	25.7 ± 10.1 (102)		82.1 (87)	
<3 BMs/week	29.7 ± 13.0 (23)	0.01	69.6 (16)	0.03
3-6 BMs/week	24.6 ± 9.0 (79)		89.9 (71)	
Male	26.0 ± 10.0 (54)	0.40	85.2 (46)	0.80
Female	25.5 ± 10.3 (48)		85.4 (41)	
≤74 years old	26.3 ± 10.5 (66)	0.21	80.1 (55)	0.64
≥75 years old	24.6 ± 9.4 (36)		84.2 (32)	

SBM: spontaneous bowel movement; PEG+E: polyethylene glycol plus electrolytes; SD: standard deviation; BM: bowel movement.

**Table 5:** Adverse events of short-duration PEG+E.

	Adverse events % (*n*)	*p* value	Abdominal pain	Increase residual stool feeling	Diarrhea	Abdominal distension	Abdominal discomfort
Overall	6.6 (7)		2	2	1	1	1
BM < 3/week	7.4 (2)	0.94	1	0	1	0	0
BM 3-6/week	6.3 (5)		1	2	0	1	1
Male	7.1 (4)	0.87	0	2	0	1	1
Female	6.0 (3)		2	0	1	0	0
≤74 years old	8.8 (6)	0.21	1	2	1	1	1
≥75 years old	2.6 (1)		1	0	0	0	0

BM: bowel movement.

## Data Availability

The patient data used to support the findings of this study are available from the corresponding author upon request. However, some of them are restricted by the institutional review board in the Nishijin Hospital.
